# 2004 Methane and Nitrous Oxide Emissions from Manure Management in South Africa

**DOI:** 10.3390/ani5020193

**Published:** 2015-03-31

**Authors:** Mokhele Edmond Moeletsi, Mphethe Isaac Tongwane

**Affiliations:** 1 ARC-Institute for Soil, Climate and Water, Private Bag X79, Pretoria 0001, South Africa; E-Mail: TongwaneM@arc.agric.za; 2 Risks and Vulnerability Assessment Centre, University of Limpopo, Private Bag X1106, Sovenga 0727, South Africa

**Keywords:** activity data, emissions factors, IPCC guidelines, manure management system

## Abstract

**Simple Summary:**

Livestock manure management is one of the main sources of greenhouse gas (GHG) emissions in South Africa producing mainly methane and nitrous oxide. The emissions from this sub-category are dependent on how manure is stored. Liquid-stored manure predominantly produces methane while dry-based manure enhances mainly production of nitrous oxide. Intergovernmental Panel on Climate Change (IPCC) guidelines were utilized at different tier levels in estimating GHG emissions from manure management. The results show that methane emissions are relatively higher than nitrous oxide emissions with 3104 Gg and 2272 Gg respectively in carbon dioxide global warming equivalent.

**Abstract:**

Manure management in livestock makes a significant contribution towards greenhouse gas emissions in the Agriculture; Forestry and Other Land Use category in South Africa. Methane and nitrous oxide emissions are prevalent in contrasting manure management systems; promoting anaerobic and aerobic conditions respectively. In this paper; both Tier 1 and modified Tier 2 approaches of the IPCC guidelines are utilized to estimate the emissions from South African livestock manure management. Activity data (animal population, animal weights, manure management systems, *etc.*) were sourced from various resources for estimation of both emissions factors and emissions of methane and nitrous oxide. The results show relatively high methane emissions factors from manure management for mature female dairy cattle (40.98 kg/year/animal), sows (25.23 kg/year/animal) and boars (25.23 kg/year/animal). Hence, contributions for pig farming and dairy cattle are the highest at 54.50 Gg and 32.01 Gg respectively, with total emissions of 134.97 Gg (3104 Gg CO_2_ Equivalent). Total nitrous oxide emissions are estimated at 7.10 Gg (2272 Gg CO_2_ Equivalent) and the three main contributors are commercial beef cattle; poultry and small-scale beef farming at 1.80 Gg; 1.72 Gg and 1.69 Gg respectively. Mitigation options from manure management must be taken with care due to divergent conducive requirements of methane and nitrous oxide emissions requirements.

## 1. Introduction 

Accurate quantification of national greenhouse gas (GHG) emissions is required to provide a sound basis of government policies and mitigation potential opportunities. Reliable information can also help in the identification of proper responses in line with food security and economic development in the country [1]*.* GHG emissions for manure management are considered as a key source category that needs to be estimated in South Africa [2]*.* Manure management includes storage and treatment of manure, before using it as fertilizer or burning as fuel. Methane (CH_4_) and nitrous oxide (N_2_O) are produced during different storage and treatment stages of manure. The term ‘manure’ includes both dung and urine produced by livestock [3]*.*

Livestock manure is primarily composed of organic material and water. Anaerobic bacteria decompose the organic material under anaerobic conditions, releasing CH_4_ [4]*.* Methane emissions from manure management are mostly associated with confined animals where manure is managed under different management systems [3,5,6]*.* The quantity of CH_4_ emitted from manure management operations is a function of three primary factors: (1) the manure management system, (2) the environmental conditions and (3) the amount and composition of the manure [4,7,8]*.* The management system determines key factors that affect CH_4_ production including contact with oxygen, water content, pH and nutrient availability [4]*.* When manure is stored or treated as a liquid in a lagoon, pond or tank it tends to decompose anaerobically and produce a significant quantity of CH_4_. In contrast, when manure is handled as a solid or deposited on pastures it tends to decompose aerobically and little or no CH_4_ is produced [3,6]*.* According to Bull *et al.* [7] and EPA [8], temperature, pH and moisture content also affect CH_4_ formation, with high temperature, high moisture level and neutral pH conditions favoring CH_4_ production. The composition of manure is directly related to animal types and diets; with dairy cattle being associated with higher feed intake and therefore higher manure excretion rates than non-dairy cattle.

Nitrous oxide is produced directly and indirectly during the storage and treatment of manure and urine. Direct emissions occur through the processes of nitrification and denitrification while indirect emissions occur through volatilization, leaching and runoff [3,6,7,9,10]*.* Nitrites and nitrates are transformed to N_2_O and dinitrogen (N_2_) during the aerobic processes of nitrification as illustrated in the following equations [6,7]:

Nitrification:


NH_4_^+^ + O_2_ → H^+^ + H_2_O + NO_2_^−^ NO_2_^−^ + O_2_ → NO_3_^−^

Denitrification:


NO_3_^−^ → NO_2_^−^ → NO → N_2_O → N_2_

Production and emission of N_2_O from manure depends on digestibility and composition of animal feed, manure management practices, duration of waste management and environmental conditions [3,11]. High N_2_O emissions are related to high intake of feed with high nitrogen concentration. N_2_O emissions depend on the amount of oxygen and moisture level of the managed manure [6,7]. Manure stored for long periods of time results in relatively high emissions of N_2_O. The environmental conditions that favor the development of N_2_O in managed manure are low pH, high temperature, increased aeration and low moisture [3,12].

In this paper, CH_4_ and N_2_O emissions from manure management are assessed utilizing the IPCC 2006 guidelines for national GHG inventory estimations. The country-specific emissions factors are calculated for animals with significant contribution to improve on the results obtained from the previous inventories.

## 2. Materials and Methods

In South Africa livestock production accounts for about 70% of the agricultural land due to an extensive area of marginal soils and low rainfall [13,14]. The climate of South Africa varies greatly across all the livestock producing areas with arid climate over the southwestern parts and mostly varying temperate and subtropical climates for the rest of the country [15]. Livestock production in South Africa varies substantially with numbers, breeds and species according to grazing, environment and production systems [commercial, small-scale or communal] [13,16]. These differences in management of livestock in the country are also evident in the livestock manure management systems which has an impact on GHG emissions from the livestock sector.

To estimate the emissions from manure management, information on how manure is stored or handled is key and this was obtained through a questionnaire addressed to experts. Thus, information on the distribution of excretion into several manure management systems (MMS) was compiled (Table 1). Dairy cattle MMS data were obtained from personal communication with dairy farm owners as well as dairy associations and managers through questionnaires. Subsistence farming cattle, beef cattle, sheep, goats, horses and donkeys MMS data were obtained through communication with Agricultural Research Council (ARC)-Animal Production Institute researchers. Pig MMS data was obtained through personal communication with ARC-Institute for Agricultural Engineering personnel and farm managers. Poultry MMS data were obtained from communication with an industrial chicken farm manager. In all these categories, 10–20 farmers, obtained from the ARC database from different production regions were interviewed, constituting less than 1% of the total number of farmers per category.

**Table 1:** Manure management system usage (%) for different livestock categories.

Livestock Category	Sub-Category	Lagoon	Liquid/Slurry	Drylot	Daily Spread	Compost	Pasture	Manure with Bedding > 1 month	Poultry Manure without Litter	Poultry Manure with Litter
Dairy cattle	Mature cows	20	5	25	0	0	45	5	0	0
	Heifers (1–2 years)	0	0	5	0	2	93	0	0	0
Commercial beef cattle	Feedlot cattle	5	5	75	5	10	0	0	0	0
	Mature cows	0	0	5	0	5	90	0	0	0
	Heifers (1–2 years)	0	0	5	0	5	90	0	0	0
	Young oxen	0	0	5	0	5	90	0	0	0
	Mature oxen	0	0	5	0	5	90	0	0	0
	Bulls	0	0	5	0	5	90	0	0	0
	Calves	0	0	5	0	5	90	0	0	0
Subsistence cattle	Mature cows	0	0	10	0	0	80	10	0	0
	Heifers (1–2 years)	0	0	10	0	0	80	10	0	0
	Young oxen	0	0	10	0	0	80	10	0	0
	Mature oxen	0	0	10	0	0	80	10	0	0
	Bulls	0	0	10	0	0	80	10	0	0
	Calves	0	0	10	0	0	80	10	0	0
Sheep	Commercial	0	0	2	0	0	98	0	0	0
	Subsistence	0	0	5	0	0	85	10	0	0
Goats	Commercial	0	0	2	0	0	98	0	0	0
	Subsistence	0	0	5	0	0	85	10	0	0
Horses		0	0	0	0	0	100	0	0	0
Donkeys		0	0	0	0	0	100	0	0	0
Pigs	Boars	50	20	20	5	5	0	0	0	0
	Sows	50	20	20	5	5	0	0	0	0
	Growers	50	20	20	5	5	0	0	0	0
Poultry	Layers	0	5	70	5	10	0	0	10	0
	Broilers	0	0	80	0	5	0	0	0	15

Methane emissions from manure management were calculated from animal population, activity and MMS data. CH_4_ emissions from cattle manure management were calculated using the Tier 2 approach, whereas emissions from manure management from all other livestock categories were estimated using the Tier 1 approach.

Table 2 shows animal weight, volatile solid excretion (VS) and maximum CH_4_-producing capacity of manure (B_o_) for all the livestock categories [3]. The VS and B_o_ values for all livestock categories were obtained from the default values of the IPCC guidelines using the Oceania values for commercial dairy, beef cattle, commercial sheep, commercial goats, pigs and poultry while Africa values were utilized for the other animal categories. For poultry, the values for developed countries were utilized. The animal weights of the commercial livestock were found to be more or less similar to the weights for Australia and New Zealand and thus the default values for these categories are from Oceania. Table 3 shows the methane conversion factors (MCF) obtained from the IPCC guidelines [3]. The annual average temperature was taken as 18 °C, which is the mean of the average temperatures from all the provinces [17].

**Table 2:** Productivity data for all the livestock sub-categories.

Livestock Category	Sub-Category	Animal Weight (kg) [1]	Volatile Solids (kg VS day^−1^)	Maximum Methane-Producing Capacity of Manure (m^3^ CH_4_ kg^−1^ of VS Excreted)
Dairy cattle	Mature cattle	498	3.50	0.24
	Heifers (1–2 years)	355	3.00	0.17
Commercial beef cattle	Feedlot cattle	300	3.00	0.17
	Mature cows	512	3.00	0.17
	Heifers (1–2 years)	331	3.00	0.17
	Young oxen	462	3.00	0.17
	Mature oxen	550	3.00	0.17
	Bulls	993	3.00	0.17
	Calves	124	3.00	0.17
Subsistence cattle	Mature cows	369	3.00	0.10
	Heifers (1–2 years)	213	3.00	0.10
	Young oxen	300	3.00	0.10
	Mature oxen	401	3.00	0.10
	Bulls	585	3.00	0.10
	Calves	85	3.00	0.10
Sheep	Commercial	69	0.40	0.19
	Subsistence	40	0.32	0.13
Goats	Commercial	50	0.30	0.18
	Subsistence	36	0.35	0.13
Horses		595	1.72	0.26
Donkeys		250	0.94	0.26
Pigs	Sows	218	0.50	0.45
	Boars	270	0.50	0.45
	Growers	80	0.28	0.45
Poultry	Layers	2.0	0.02	0.39
	Broilers	1.8	0.01	0.36

**Table 3:** Intergovernmental Panel on Climate Change (IPCC) 2006 default methane conversion factors for different manure management systems.

Lagoon	Liquid/Slurry	Drylot	Daily Spread	Compost	Pasture	Manure with Bedding >1 month	Poultry Manure without Litter	Poultry Manure with Litter
77	35	1.5	0.5	0.5	1.5	35	1.5	1.5

Nitrous oxide emissions from manure management were calculated from animal population data, activity data and MMS data. Table 4 shows animal weight, nitrogen excretion rate (N_rate_), and annual N excretion per head of livestock (N_ex_) for all livestock categories. The N_rate_ was obtained from the Oceania default values for dairy cattle, commercial beef cattle, commercial sheep, commercial goats, pigs and poultry while Africa IPCC default values were utilized for subsistence livestock, horses and donkeys. The N_ex_ was estimated using equation 10.30 from the IPCC guidelines [3].

**Table 4:** Activity data, per livestock category, required for calculating N_2_O emissions from manure management in 2004.

Livestock Category	Sub-Category	Animal Weight (kg) [1]	Nitrogen Excretion Rate (kg N (1000 kg·animal·mass)^−1^·d^−1^)	Annual Nitrogen Excretion per Head (kg·N·animal^−1^·year^−1^)
Dairy cattle	Mature cattle	498	0.44	79.98
	Heifers (1–2 years)	355	0.50	63.88
Commercial beef cattle	Feedlot cattle	300	0.50	54.75
	Mature cows	512	0.50	93.44
	Heifers (1–2 years)	331	0.50	60.41
	Young oxen	462	0.50	84.32
	Mature oxen	550	0.50	100.38
	Bulls	993	0.50	181.22
	Calves	124	0.50	39.97
Subsistence cattle	Mature cows	369	0.63	84.85
	Heifers (1–2 years)	213	0.63	48.98
	Young oxen	300	0.63	68.99
	Mature oxen	401	0.63	92.21
	Bulls	585	0.63	134.52
	Calves	85	0.63	19.55
Sheep	Commercial	69	1.13	28.46
	Subsistence	40	1.17	17.08
Goats	Commercial	50	1.42	25.92
	Subsistence	36	1.37	18.00
Horses		595	0.46	99.90
Donkeys		250	0.46	41.98
Pigs	Sows	218	0.46	36.60
	Boars	270	0.46	45.33
	Growers	80	0.53	15.48
Poultry	Layers	2	0.82	0.60
	Broilers	1.8	1.10	0.72

## 3. Results and Discussion

**Figure 1:**
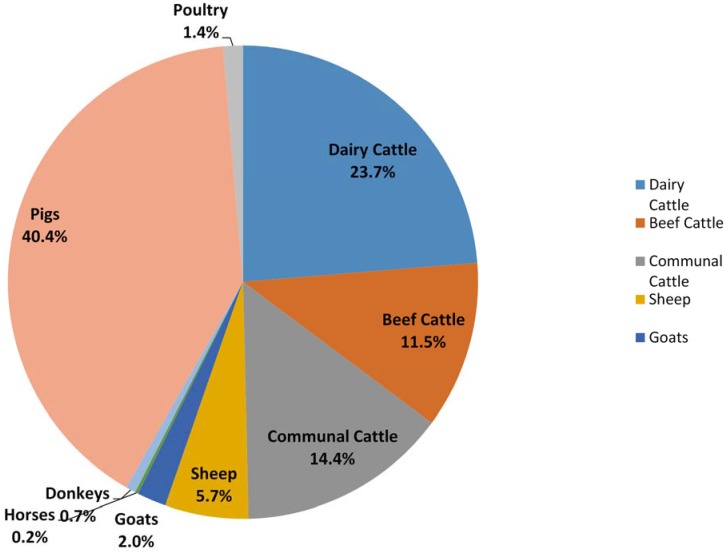
CH_4_ emissions from manure management per livestock category in 2004.

Mature female dairy cows have the highest CH_4_ emissions factor with 40.98 kg/year (Table 5). This is followed by sows and boars both at 25.23 kg/year while the other animal sub-categories had emissions factors below 10 kg/year. The emissions factors calculated for sheep, goats, horses, donkeys and poultry are the same as those found in the IPCC guidelines default tables (IPCC, 2006). The total CH_4_ emissions from direct manure management are estimated at 134.97 Gg (3104 Gg CO_2_ Equivalent) (Table 5) with pig industry, dairy cattle and small-scale cattle farming showing the highest emissions of 54.5 Gg (40.4%), 32.01 Gg (23.7%) and 19.50 Gg (14.4%) respectively (Figure 1). These relatively high emissions are attributed to MMS which are perceived to be based on slurry, cattle bedding and lagoons. The lowest emissions are from poultry, donkeys and horses with less than 2.00 Gg each.

Due to lack of country-specific activity data on manure characteristics, the Tier 1 approach had to be used for some of the animal sub-categories and thus there is a lot of uncertainty associated with the emissions estimated. Data on manure management storage systems under different livestock categories is lacking, with estimates being used based on expert opinions. Uncertainty on manure management is therefore high. A country average temperature was used and this leads to inaccuracies in the estimates as some of the MMS (e.g., liquid/slurry systems) are highly sensitive to temperature variations. To reduce this uncertainty, the percentage of animal populations, and thus manure management systems, in different temperature zones needs to be determined so that a more specific MCF can be used and a weighted average emissions factor can be determined.

**Table 5:** Methane emissions from manure management (main category totals in bold).

Livestock Category	Sub-Category	Animal Population	Emissions Factor	Tier Level	CH_4_ Emissions (Gg)
Dairy cattle	Mature cattle	770,000	40.98	2	31.55
	Heifers (1–2 years)	250,000	1.85	2	0.46
		**32.01**			
Commercial beef cattle	Feedlot cattle	420,000	8.48	2	3.56
	Mature cows	2,840,000	1.81	2	5.14
	Heifers (1–2 years)	1,390,000	1.81	2	2.52
	Young oxen	520,000	1.81	2	0.94
	Mature oxen	280,000	1.81	2	0.51
	Bulls	200,000	1.81	2	0.36
	Calves	1,350,000	1.81	2	2.44
		**15.47**			
Subsistence cattle	Mature cows	2,223,314	3.56	2	7.91
	Heifers (1–2 years)	1,088,171	3.56	2	3.87
	Young oxen	407,086	3.56	2	1.45
	Mature oxen	219,200	3.56	2	0.78
	Bulls	156,571	3.56	2	0.56
	Calves	1,385,657	3.56	2	4.93
		**19.50**			
Sheep	Commercial	22,289,000	0.28	1	6.24
	Subsistence	3,070,000	0.49	1	1.50
		**7.74**			
Goats	Commercial	2,164,000	0.20	1	0.43
	Subsistence	4,224,000	0.54	1	2.28
		**2.71**			
Horses		180,000	1.64	1	**0.3**
Donkeys		1,000,000	0.90	1	**0.9**
Pigs	Sows	1,559,000	25.23	1	39.33
	Boars	91,000	25.23	1	2.30
	Growers	910,356	14.13	1	12.87
		**54.50**			
Poultry	Layers	17,590,000	0.06	1	1.06
	Broilers	77,561,644	0.01	1	0.78
					**1.84**			
**Total CH_4_ emissions from manure management**					**134.97**

The data gathered on methane emissions from MMS for different livestock in 2004 differ from the 1990 inventory (Table 6). In 2004 the amount of manure which dairy and pig farmers stored in a liquid form was estimated to be greater than it was in 1990, hence the much higher emissions [18]. Furthermore, dairy cattle had a higher emissions factor and pigs had a greater population number (due to the inclusion of growers) than the 1990 inventory, hence the higher emissions for these sub-categories. Emissions from beef cattle manure management were reduced in 2004, mainly due to the lower emissions factor (1.81) (commercial) in 2004 compared to 3.62 used in 1990/2000 [2,18]. Even though the poultry number is much increased, emissions are down from 1990 due to a much reduced emissions factor.

**Table 6:** Comparison of CH_4_ emissions from manure management in 2004 with previous inventories.

Livestock Category	1990		2000		2004	
	Population	CH_4_ Emissions (Gg)	Population	CH_4_ Emissions (Gg)	Population	CH_4_ Emissions (Gg)
Dairy cattle	840,000	4.31	846,000	4.34	1,020,000	32.01
Beef cattle	12,660,000	45.83	12,754,000	46.16	12,479,683	34.97
Goats and sheep	37,172,000	8.55	35,257,000	8.11	31,747,000	10.45
Horses	770,000	1.24	270,000	0.43	180,000	0.30
Donkeys	150,000	0.24	150,000	0.24	1,000,000	0.90
Pigs	1,532,000	17.19	1,556,000	17.45	2,560,356	54.50
Poultry	51,787	6.05	119,000	13.92	95,151,644	1.84
**Total**		**83.41**		**90.65**		**134.97**

Table 7 shows that all other MMS have N_2_O emissions factors of zero with the exception of drylot, compost, manure with bedding and poultry manure with/without litter with 0.02, 0.01, 0.01 and 0.001 respectively [3].

**Table 7:** IPCC 2006 default N_2_O emissions factors for different manure management systems.

Lagoon	Liquid/Slurry	Drylot	Daily Spread	Compost	Pasture	Manure with Bedding > 1 month	Poultry Manure without Litter	Poultry Manure with Litter
0	0	0.02	0	0.01	0	0.01	0.001	0.001

**Figure 2:**
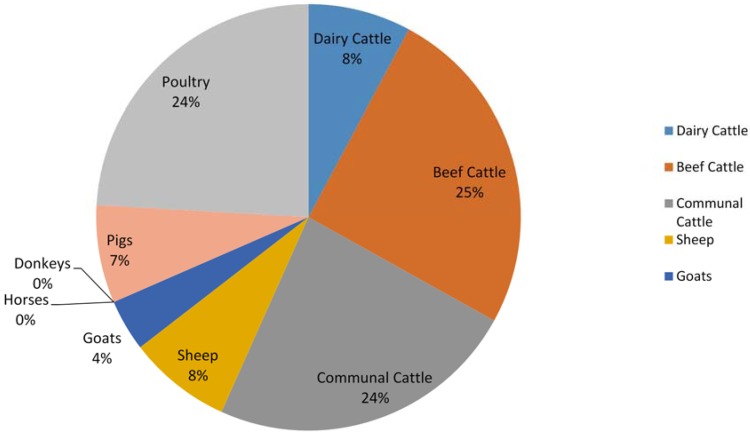
N_2_O emissions from manure management per livestock category for 2004.

The total estimated direct N_2_O emissions from manure management add up to 7.10 Gg (2272 Gg CO_2_ Equivalent). N_2_O emissions from manure management were calculated for each livestock category, with commercial beef cattle contributing the most at 1.80 Gg, constituting 25% of the total N_2_O emissions (Figure 2 and Table 8). The other main contributors are poultry farming and subsistence cattle farming with 1.72 Gg (24%) and 1.69 Gg (24%) respectively. Due to the fact that emissions factors for the lagoon, pasture and liquid/slurry MMS are all zero, emissions from horses and donkeys are infinitesimal while emissions from dairy cattle and pig farming are insignificant.

The weights used for different animal categories were mostly based on average weights from specific species, hence the data variance is high. The default nitrogen excretion rate values were used for Oceania and these had an uncertainty of ±50%. Default values were used to determine the N_2_O emissions from manure management and thus no data comparisons were made.

The total N_2_O emissions from manure management in 2004 are much higher (7.10 Gg) than the 1990 emissions (1.34 Gg) [18]. In the 1990 inventory the data were not given per livestock category but rather by MMS. All the N_2_O emissions in 1990 were from drylot and solid storage management systems, while in 2004 data gathered shows an array of different MMS was utilized by the farmers. There are several differences between the 1990 and 2004 inventories which contribute to the increased N_2_O emissions. Firstly, there was the difference in the MMS data with the 2004 inventory having a much higher percentage of manure being handled in drylot than previously suggested. Secondly, the nitrogen excretion values used in the 1990 inventory were much lower than those calculated (using IPCC default values) for the 2004 inventory. It is not clear from where the nitrogen excretion rates for the 1990 inventory were obtained, making it difficult to assess the possible reasons for this discrepancy. Thirdly, the animal population numbers were different.

**Table 8:** N_2_O emissions from manure management per livestock sub-category for 2004.

Livestock Category	Sub-Category	Animal Population	N_2_O Emissions (Gg)
Dairy cattle	Mature cattle	770,000	0.53
	Heifers (1–2 years)	250,000	0.03
	**0.56**		
Commercial beef cattle	Feedlot cattle	420,000	0.58
	Mature cows	2,840,000	0.63
	Heifers (1–2 years)	1,390,000	0.20
	Young oxen	520,000	0.10
	Mature oxen	280,000	0.07
	Bulls	200,000	0.09
	Calves	1,350,000	0.13
	**1.80**		
Subsistence cattle	Mature cows	2,223,314	0.89
	Heifers (1–2 years)	1,088,171	0.25
	Young oxen	407,086	0.13
	Mature oxen	219,200	0.10
	Bulls	156,571	0.10
	Calves	1,385,657	0.22
	**1.69**		
Sheep	Commercial	22,289,000	0.40
	Subsistence	3,070,000	0.16
	**0.56**		
Goats	Commercial	2,164,000	0.04
	Subsistence	4,224,000	0.24
	**0.28**		
Horses		180,000	**0**
Donkeys		1,000,000	**0**
Pigs	Sows	1,559,000	0.40
	Boars	91,000	0.03
	Growers	910,356	0.10
	**0.53**		
Poultry	Layers	17,590,000	0.25
	Broilers	77,561,644	1.47
	**1.72**		
**Total N_2_O emissions from manure management**			**7.10**

## 4. Conclusions

Livestock manure in South Africa is mostly left in the pasture, range or paddocks or managed as drylot and the CH_4_ conversion factor in these systems is low (1.5%). Dairy cattle and pig manure are slightly different in that a higher percentage of the manure is managed in a liquid form. These management systems have much higher MCFs, therefore dairy cattle and pig manure management made the greatest contribution (32.01 Gg {736 Gg CO_2_ Equivalent} and 54.5 Gg {1254 Gg CO_2_ Equivalent} respectively) to the total CH_4_ manure management emissions in 2004. On the other hand, no N_2_O is produced from manure managed in lagoon, liquid/slurry, daily spread and pastures, hence emissions from dairy cattle and pig farming are relatively low. The main emitters in this category are commercial beef cattle, poultry and subsistence cattle farming with around 25% contribution per category.

In order to improve the accuracy and reduce the uncertainty of the manure management emissions data it is very important to enhance the monitoring of MMS. The manure management usage data is solely based on expert opinion. The other improvement would be to obtain information on the percentage of animal populations in different temperature zones, or even provincial data, so that a more accurate weighted average emissions factor can be determined. N_2_O emissions data from MMS would also be improved if nitrogen excretion rates for cattle in South Africa were determined so that actual data could be used instead of the value calculated using IPCC default values.

Mitigation of manure management emissions is crucial towards tackling the impacts of climate change. But policymakers and implementers should carefully balance prospective mitigation options for the country and region, taking note of the fact that conditions for CH_4_ and N_2_O emissions in MMS are contradictory. Issues of emissions factors of different MMS, regional or national distributions of manure in different MMS, and global warming potential of both CH_4_ and N_2_O must play an important role in the choice of mitigation options.
